# Investigating bidirectional causality between prostate cancer and inflammatory factors: A 2-sample Mendelian randomization analysis

**DOI:** 10.1097/MD.0000000000044180

**Published:** 2025-09-05

**Authors:** Jieyan Wang, Qi Cheng, Fangyu Luo, Yantong Wan, Hui Liang

**Affiliations:** a Department of Urology, The People’s Hospital of Longhua, Shenzhen, China; b College of Second Clinical Medicine, Southern Medical University, Guangzhou, China; c College of Traditional Chinese Medicine, Southern Medical University, Guangzhou, China; d Department of Pathophysiology, Guangdong Provincial Key Laboratory of Proteomics, School of Basic Medical Sciences, Southern Medical University, Guangzhou, China.

**Keywords:** biomarkers, GWAS, inflammation, Mendelian randomization, prostate cancer

## Abstract

Growing evidence have indicated the bidirectional relationships between various inflammatory cytokines and prostate cancer (PCa), but the causality between genetic susceptibility to inflammatory cytokines and PCa was still in initial exploratory phase. This bidirectional Mendelian randomization (MR) research was manipulated to draw causative inferences and the effect of direction between 91 inflammatory cytokines and PCa. Genetic data of PCa were originated from a publicly accessible genome-wide association study with 3269 individuals and 459,664 controls, and inflammatory cytokines summarized by a protein quantitative trait locus study were embodied 14,824 participants. We considered inverse variance weighted as a primarily statistical approach, and utilized MR-Egger regression, weighted median, MR-PRESSO, and simulation extrapolation method to enhance the accuracy of the ultimate outcome. In sensitivity analysis, MR-Egger method and Cochran *Q* statistic of inverse variance weighted were employed to access the heterogeneity. The results suggested a causal relationship between fms-related tyrosine kinase 3 ligand (Flt3L), recombinant monocyte chemotactic protein (MCP) 2, MCP4, and the incidence of PCa (odds ratio [OR]: 1.0016, 95% confidence interval [CI]: 1.0000–1.0032, *P* = .045; OR: 0.9979, 95% CI: 0.9958–1.0000, *P* = .045; OR: 1.0012, 95% CI: 1.0001–1.0023, *P* = .031). In addition, reverse analysis showed that PCa was correlated with the elevated level of adenosine deaminase, axin-1, C-X-C motif chemokine ligand 6, Flt3L, interleukin (IL)-24, and IL-33 (Beta: 1.7661, 95% CI: 0.2092–3.3229, *P* = .026; Beta: 1.9185, 95% CI: 0.1548–3.6822, *P* = .033; Beta: 1.9681, 95% CI: 0.4207–3.5155, *P* = .013; Beta: 1.6589, 95% CI: 0.0733–3.2446, *P* = .040; Beta: 2.2091, 95% CI: 0.4682–3.9500, *P* = .013; Beta: 1.8438, 95% CI: 0.0815–3.6061, *P* = 040). This study highlighted the causality between several inflammatory factors and the setting of PCa. Specifically, the results suggested that Flt3L and MCP4 may be risk factors for PCa, whereas MCP2 may be a favorable factor for PCa. Conversely, adenosine deaminase, axin-1, C-X-C motif chemokine ligand 6, IL-24, IL-33, and Flt3L were involved in the downstream of PCa progression.

## 1. Introduction

Prostate cancer (PCa) is the second most male solid-organ malignant tumors worldwide,^[1]^ with a global age standardized incidence rate of approximately 30.7 cases per 100,000 males and the age standardized mortality rate for PCa worldwide is 7.7 cases per 100,000 people.^[2]^ The main type of PCa is adenocarcinoma (95%), followed by small cell carcinoma, which has a high metastatic rate and intertumoral heterogeneity.^[3,4]^ Early stage prostate cancer (PCa) often has no obvious symptoms, while late stage can cause disabilities such as bone pain and difficulty urinating. The exact etiology and pathogenesis of PCa are associated with several reasons and still uncertain.^[5,6]^ High-frequency gene fusion caused by multi genomic variations is considered one of the pathogenesis mechanisms of PCa.^[7]^ Chronic inflammation can mediate alterations in gene expression profiles and inflammatory response regulation via various signaling pathways, creating a favorable microenvironment for the invasion and metastasis of PCa.^[8]^

Inflammatory factors exert pleiotropic immune regulation in the pathogenesis of PCa. The several certain inflammatory factors can facilitate immune escape of tumor cells and accelerate tumor proliferation.^[9,10]^ Meanwhile, specific cytokines may take anti-inflammatory actions and may inhibit cancer deterioration. Previous studies summarized that the high expression of inflammatory factors, including tumor necrosis factor (TNF), interleukin (IL)-6, IL-18, and nuclear factor Kappa B are correlated with the mechanism of prostate carcinogenesis and exacerbation.^[11,12]^ However, factors like IL-10 and IL-18 have been confirmed to cause apoptosis of PCa tumor cells and have protection on premalignant lesion.^[13]^ The biological functions of inflammation in the pathogenesis and subsequent infection of PCa remain controversial. The traditional treatment methods for PCa are mainly radical surgery and endocrine therapy, which all have certain degrees of side effects. In clinical oncology, immunotherapy has emerged as an indispensable and highly effective therapeutic modality, revolutionizing treatment outcomes for select cancers.^[14]^ Immunosuppressive agents commonly used in the treatment of PCa may have ototoxicity and other malpractice, so it is necessary to find new therapeutic targets.^[15]^

Mendelian randomization (MR) is an epidemiological approach for assessing and quantifying causality by exploiting genetic variation as instrumental variables (IVs). This method is based on analyzing large samples of gene and phenotype data, minimizing confounding factors, and bias in reverse causality, thereby promoting more accurate causal ratiocination.^[16]^ In this study, we capitalized on single nucleotide polymorphisms (SNPs) of 91 inflammatory cytokines and PCa as IVs stemmed from 2 different public genome-wide association study (GWAS) databases, conducting bidirectional MR analysis and elucidating the potential causal relationships in this nexus. The analysis results provide reliable bioinformatics basis for the study of the etiopathogenesis and subsequent effects in PCa.

## 2. Method

### 2.1. MR assumptions

**Figure 1.**
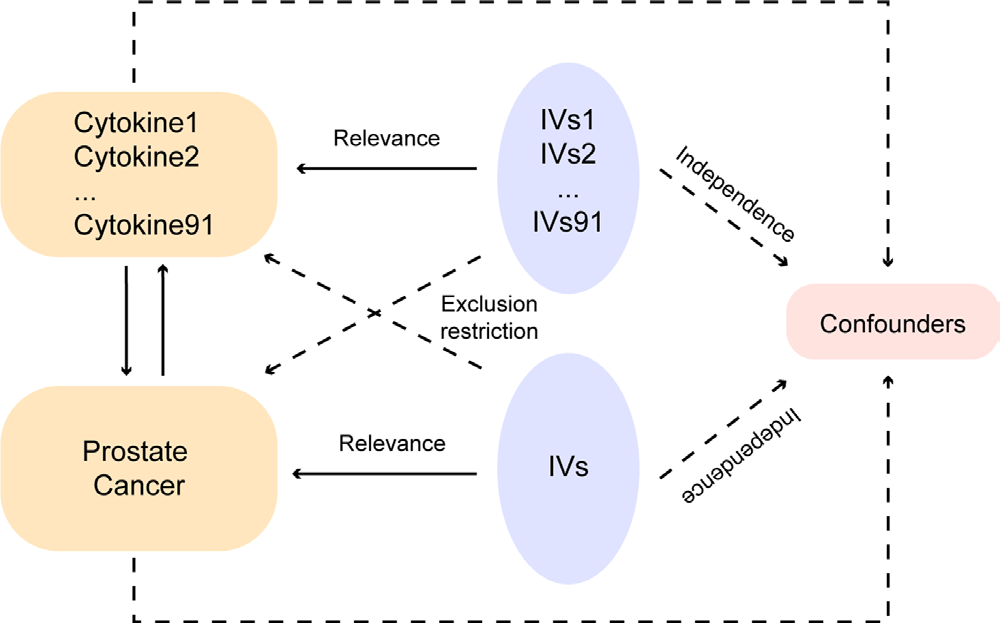
Schematic of the study design in this bidirectional Mendelian randomization analysis. Significant instrumental variables were selected for 91 inflammatory cytokines and PCa. Three basic assumptions of MR analysis were illustrated in this graph, namely, relevance, independence, and exclusion restrictions. PCa = prostate cancer.

In this MR analysis, we integrated plasma proteomic datasets of 91 inflammatory cytokines and genomic datasets from 2 polycentric observational study to comprehensively estimate the relevance between PCa and inflammatory factors from forward and reverse directions (Fig. 1). The selection of IV mainly based on 3 critical premises^[17]^: independence premise: IVs have no correlation with all confounders; correlation premise: IVs have high relativity with exposures; exclusivity premise: IVs only affects results through exposure elements.^[18]^ The GWAS dataset used in this epidemiological analysis is publicly available and had undergone ethical review, hence no additional informed consent was required. This MR analysis was conducted under the guidance of the STROBE-MR statement.^[16]^

### 2.2. Data source

Two datasets were utilized from publicly available summary GWAS data. In this MR analysis, the plasma proteomic datasets of 91 inflammatory factors were collected from a large-scale protein quantitative trait locus GWAS, enlisting 11 cohorts with 14,824 participants of Europeans.^[19]^ The genome-wide genetic data of PCa was obtained from a GWAS database in UK Biobank (https://www.ukbiobank.ac.uk), including 3269 cases and 459,664 controls of European origin. The selection of demographic for the exposures and outcomes was inconsistent.

### 2.3. Selection of IVs

To filter the closely correlated SNPs to inflammatory cytokines and PCa as IVs, *P* > 5 × 10^−8^ was considered statistically significant in Bonferroni correction. Since few SNPs were identified in partial cytokines at the time of exposure, a higher threshold value (*P* < 5 × 10^-6^) was opted to obtain more genotype records and guarantee the authenticity and effectiveness of the conclusion. Window size = 10,000 kb and *r*^2^ ≤ 0.1 were set to screen out independent SNPs and eliminate linkage disequilibrium among IVs.^[19]^ Palindromic SNPs were abandoned by MR-PRESSO, in order to avoid horizontal pleiotropy and ensure that there are no identical alleles among 91 inflammatory cytokines and PCa. *R*^2^ value, representing the level of exposure explained by IVs, were applied to assess the proportion of variance.^[20]^

### 2.4. Statistical analysis

This study applied the inverse variance weighted (IVW) method as the main analysis method, and employed the MR-Egger approach and weighted median (WM) approach as supplementary statistical approaches. By calculating *P*-values and odds ratios (ORs) values, the consistency of results from different MR analysis methods is determined to enhance the robustness of causal relationships. IVW weighted the causal effects of traits through different genetic variations, and then combines the weighted estimated effects to estimate the causal effects of genes on traits. MR-Egger regression identified the pleiotropy of SNP levels and corrected for multiple effects, improving the accuracy of causal relationship estimation.^[21]^ The MR-Egger (simulation extrapolation [SIMEX]) method could be performed to fix attenuation deviation. The WM method mainly assigns different weights to different genetic variations, thereby reducing the impact of extreme genetic variations on causal inference. MR-PRESSO was employed to eliminate outliers and distinguish IVs with horizontal pleiotropy by SNP specific detection.^[22]^ Heterogeneity of the MR-Egger and IVW model from each SNP were determined by Cochran *Q* statistic, and heterogeneity was considered to be present when *P* < .05, at which point causal inference was made using the random effects model of IVW.

In forward MR analysis, the causal assessments were expressed in homologous 95% confidence intervals (CIs) and ORs, while log10 ORs were utilized in inversion analysis. Considering the large quantity of cytokines investigated, Bonferroni correction was performed to circumvent errors caused by various analytical approaches.^[23]^ *P* < .00055 was supposed to have statistical significance (Bonferroni correction with 91 adjustments). Subsequently, the leave-one-out method was conducted as a sensitivity analysis to evaluate the impact of each SNP on the IVW results and detect confounders, by excluding a single SNP each time in turn.^[24]^ Two-sample MR package and MR-PRESSO package^[25]^ in the statistical software R (version 4.1.2) developed by the R Foundation for Statistical Computing (Vienna, Austria; https://www.r-project.org/) were utilized in all data analyses of this bidirectional MR study.

## 3. Results

### 3.1. Influence of 91 inflammatory cytokines on PCa

The primary MR analysis method for each of the 91 inflammatory cytokines IVs was assigned in accordance with the predetermined standard. When the threshold for genome-wide significance value was set to 5 × 10^-6^, IVs from a protein quantitative trait locus mapping of 91 inflammatory cytokines were extracted for sequential studies. After removing weak IVs, the SNPs of inflammatory cytokines and PCa complied with the independence and exclusivity assumptions, suggesting that weak instrument deviation was implicit. Following the exclusion of abnormal SNPs verified via MR-PRESSO, none of the IVs exhibited heterogeneity (Tables S1–S3, Supplemental Digital Content, https://links.lww.com/MD/P839).

The hypothesis testing of 91 inflammatory cytokines was mainly examined by means of IVW method, except for specific proteins encompassing eukaryotic translation initiation factor 4E binding protein 1, C-X-C motif chemokine ligand (CXCL) 9, eotaxin, interleukin 10 receptor subunit beta (IL-10RB), IL-12B, IL-15RA, IL-1a, monocyte chemotactic protein (MCP) 3, matrix metallopeptidase 10, tumor necrosis factor B (TNFB), and TNF receptor superfamily member 9 (TNFRSF9). The *P*-values of IVW and MR-Egger *Q*-test for these 11 inflammatory cytokines were below 0.05, hence the MR-Egger (SIMEX) method was used as an alternative statistical analysis method. After excluding abnormal SNPs authenticated by MR-PRESSO, there was no expression of heterogeneity for any of the IVs. (Table S2, Supplemental Digital Content, https://links.lww.com/MD/P839).

**Figure 2.**
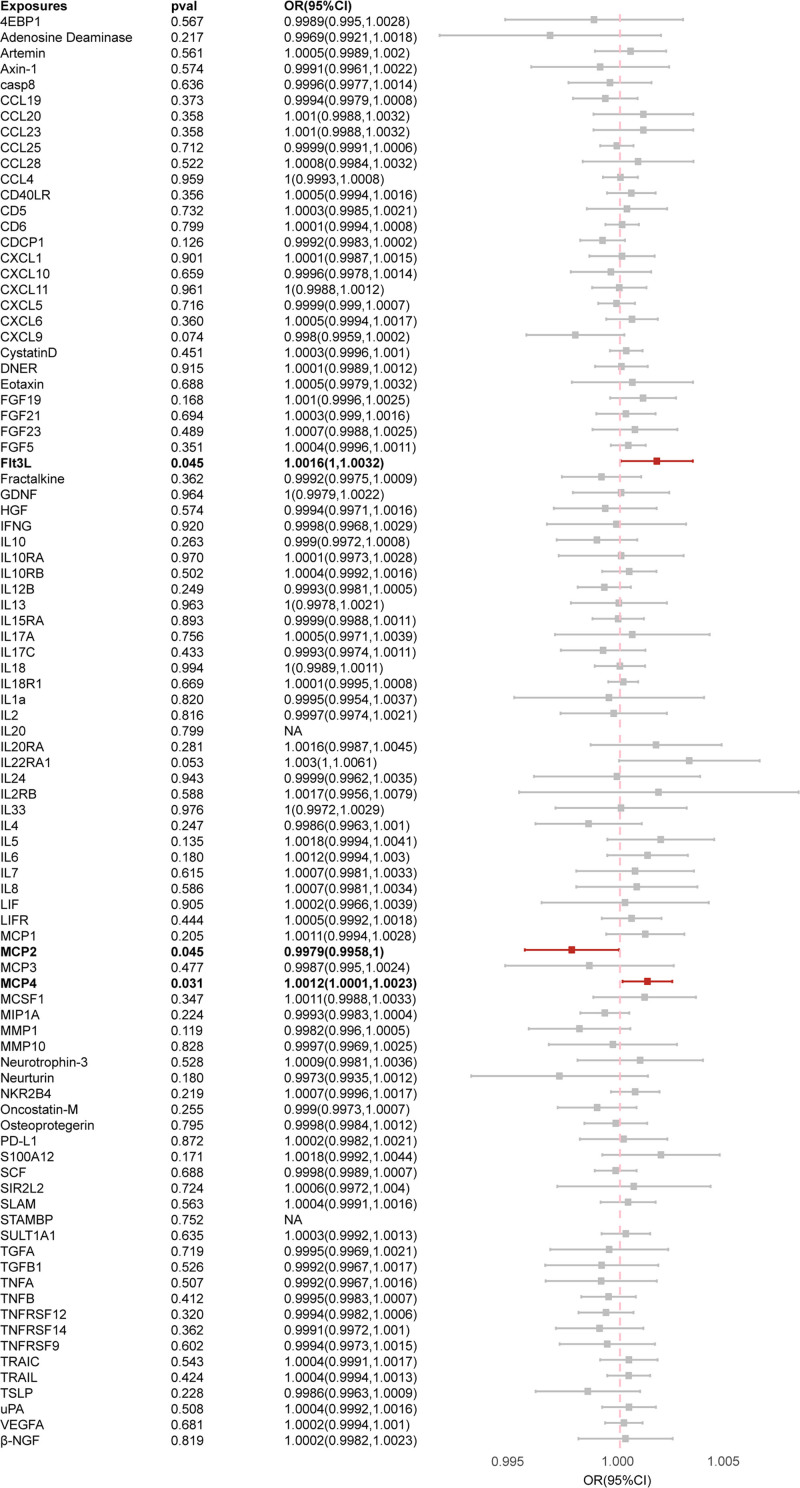
Causal correlations of 91 inflammatory cytokines on PCa. The change in the ORs of PCa per 1-SD rise in the cytokine level is shown by OR and 95% confidence interval. *P*-value .05/91 = 0.00055 was found significant after multiple-comparison correction. The results from IVW method were shown for all cytokines, except for specific proteins encompassing eukaryotic translation initiation factor 4EBP1, CXCL9, eotaxin, IL-10RB, IL-12B, IL-15RA, IL-1a, MCP3, MMP10, TNFB, TNFRSF9 wherein MR-Egger (simulation extrapolation) was considered as the recommended method. CXCL = C-X-C motif chemokine ligand, 4EBP1 = 4E binding protein 1, IL = interleukin, IVW = inverse variance weighted, MCP = monocyte chemoattractant protein, MMP = matrix metallopeptidase, MR = Mendelian randomization, ORs = odds ratios, PCa = prostate cancer, TNF = tumor necrosis factor, TNFRSF12 = TNF receptor superfamily, WM = weighted median.

The major MR analysis results characterizing causality between inflammatory biomarkers and PCa were elucidated in Figure 2 and Table S1, Supplemental Digital Content, https://links.lww.com/MD/P839. Outcomes of the IVW approach revealed an inverse correlation between PCa and MCP2 (OR: 0.9979, 95% CI: 0.9958–1.0000, *P* = .045). Similarly, consistent results were achieved across WM methods, demonstrating the negative relevance between MCP2 and PCa morbidity. Conversely, the IVW method verified positive associations between PCa and 2 cytokines, namely fms-related receptor tyrosine kinase 3 ligand (Flt3L) (OR: 1.0016, 95% CI: 1.0000–1.0032, *P* = .045) and MCP4 (OR: 1.0012, 95% CI: 1.0001–1.0023, *P* = .031), and analogous conclusions were surveyed in WM approach and RAPS approach, further supporting the positive association between MCP4 and PCa incidence. Although a statistically significant correlation in the WM method between Flt3L and PCa was undiscovered, it validated similar tendencies and yielded an almost identical result.

**Figure 3.**
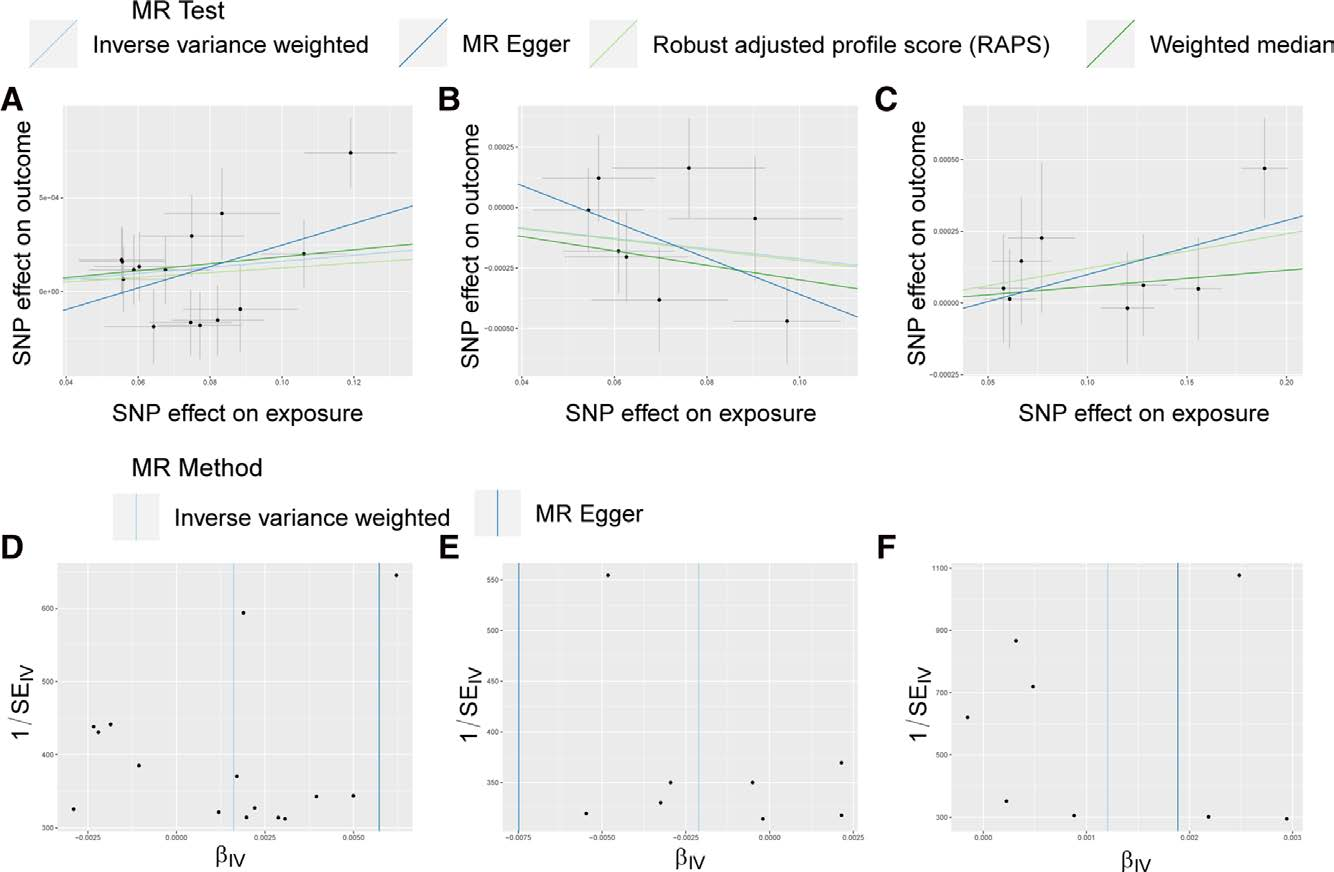
Scatter plots and funnel plots of MR analyses for Flt3L, MCP2, and MCP4 in PCa. (A–C) Individual inverse variance (IV) associations with cytokine risk are displayed versus individual IV associations with PCa in black dots. The 95% CI of odd ratio for each IV is shown by vertical and horizontal lines. The slope of the lines represents the estimated causal effect of the MR methods. (D–F) The funnel plots show the IVW MR estimate of each cytokine single nucleotide polymorphism with PCa versus 1/standard error (1/SEIV). CIs = confidence intervals, Flt3L = fms-related receptor tyrosine kinase 3 ligand, MCP = monocyte chemoattractant protein, PCa = prostate cancer.

Further sensitivity analysis was conducted on the significant causal relationship adjusted for Bonferroni, and Cochran *Q* test showed no heterogeneity in the causal relationship between Flt3L, MCP2, MCP4, and PCa. The leave-one-out analyses showed that no individual SNPs had an impact on the overall causal estimation (Figure S1, Supplemental Digital Content, https://links.lww.com/MD/P838). The distribution of causal effects shown in the funnel plot is basically symmetrical, indicating that the results are not biased by potential confounding factors. Scatter plots and funnel plots of MR analyses of Flt3L, MCP2, and MCP4 in PCa are shown in Figure 3.

### 3.2. Influence of PCa on 91 inflammatory cytokines

The significant SNPs associated with PCa from a comprehensive GWAS were selected as IVs for exploration of the causality between inflammatory factors and PCa. A critical value of *P* < 5 × 10^-8^ was installed for the option of the IVs, and the IVW approach was regarded as a primary analytical method for substantial part of cytokines, exclusive of C-C motif chemokine ligand 23, C-C motif chemokine ligand 25, glial cell line-derived neurotrophic factor, IL-17C, MCP1, sec1 family, and TNFRSF12. The selected inflammatory factors were substitutable to analyze the data via MR-Egger (SIMEX) method (Tables S4–S6, Supplemental Digital Content, https://links.lww.com/MD/P839).

**Figure 4.**
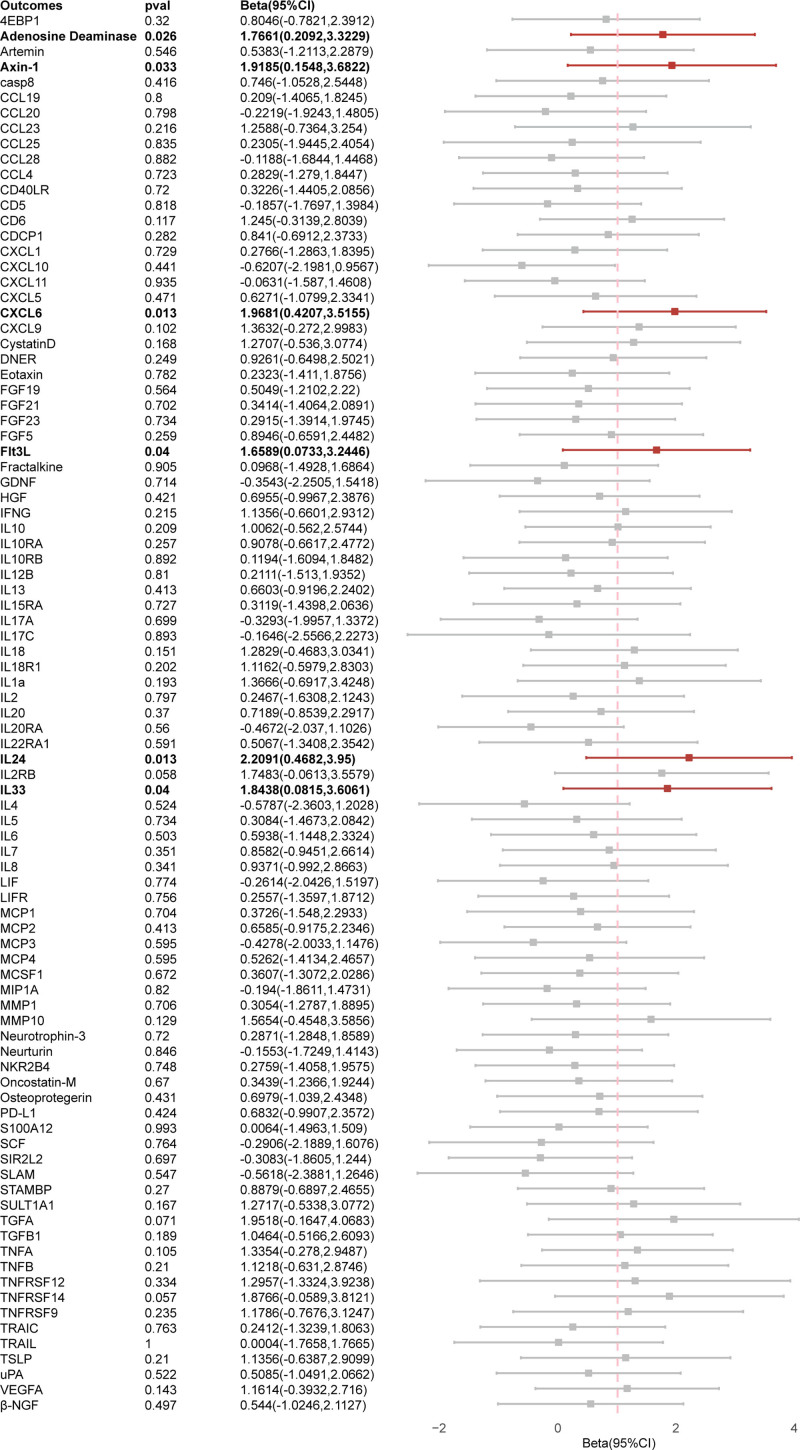
Causal correlations of PCa on 91 inflammatory cytokines. The change in the SD of inflammatory cytokines per log odds increase in PCa is represented by beta and the 95% confidence interval. *P*-value .05/91 = .00055 was found significant after multiple-comparison correction. The results from IVW method were shown for all cytokines, except for CCL23, CCL25, GDNF, IL-17C, MCP1, SCF, TNFRSF12 wherein MR-Egger (simulation extrapolation) was considered as the recommended method. CCL = C-C motif chemokine ligand, GDNF = glial cell derived neurotrophic factor, IL = interleukin, MCP = monocyte chemoattractant protein, MR = Mendelian randomization, PCa = prostate cancer, SCF = stem cell factor, TNFRSF12 = TNF receptor superfamily.

**Figure 5.**
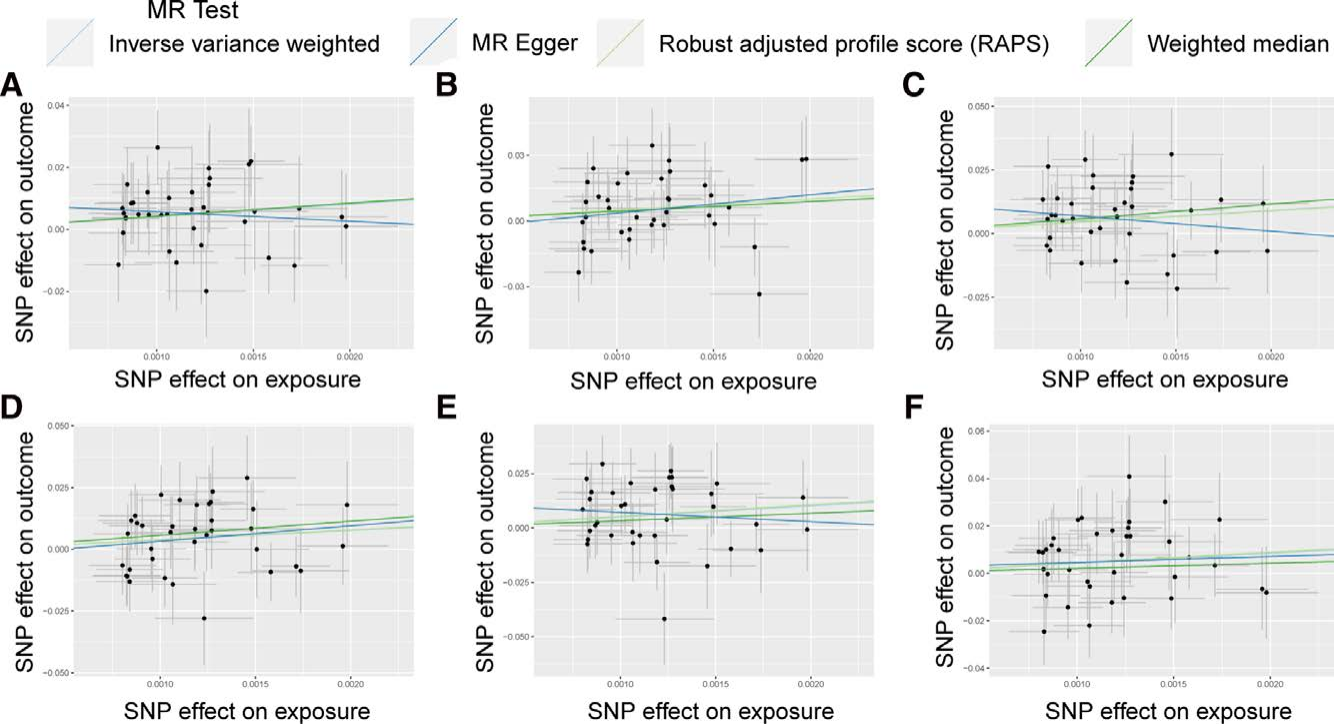
Scatter plots of MR analyses between PCa and inflammatory cytokines. Individual IV associations with PCa risk are displayed versus individual IV associations with cytokines in black dots. The 95% CI of the ORs for each IV is shown by the vertical and horizontal lines. The slope of the lines represents the estimated causal effect of the MR methods. (A–F): Adenosine deaminase, axin-1, CXCL6, Flt3L, IL-24, and IL-33. CIs = confidence intervals, CXCL = C-X-C motif chemokine ligand, Flt3L = fms-related receptor tyrosine kinase 3 ligand, IV = instrumental variable, IL = interleukin, MR = Mendelian randomization, ORs = odds ratios, PCa = prostate cancer.

**Figure 6.**
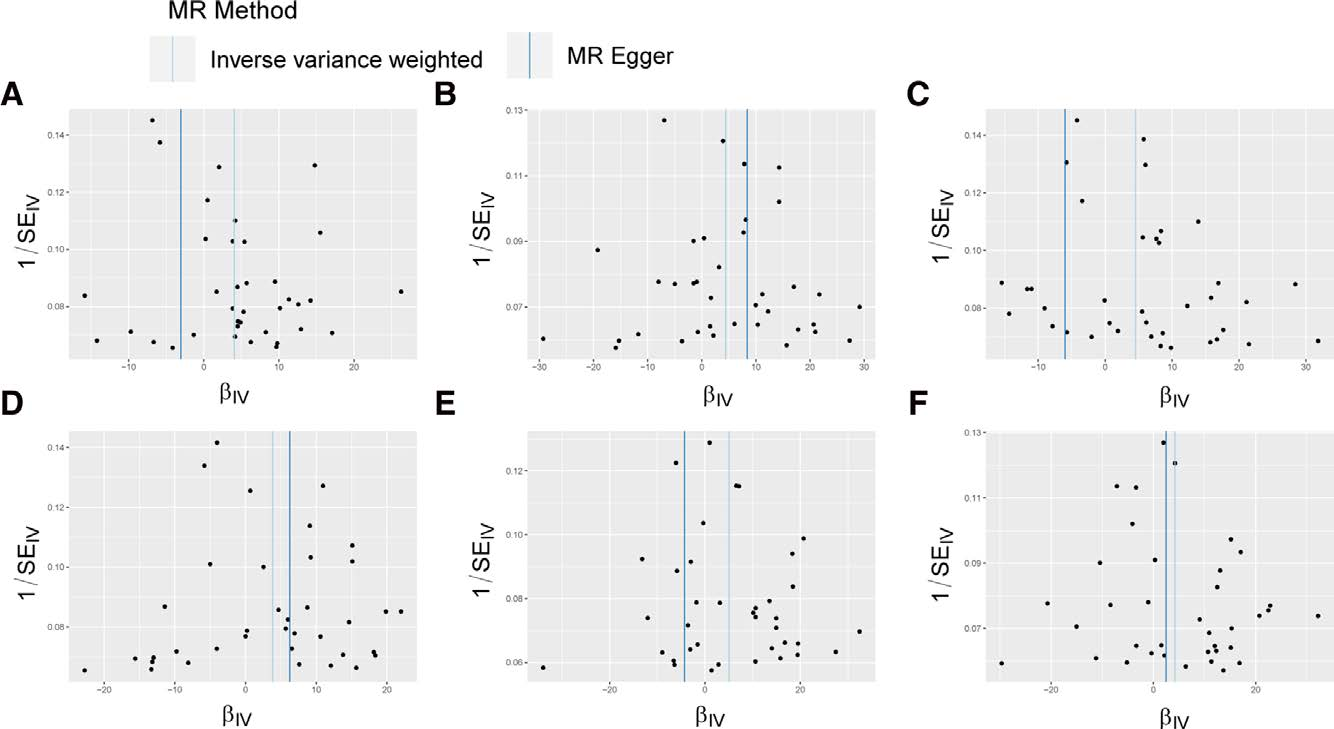
Funnel plots of MR analyses between PCa and inflammatory cytokines. The funnel plots show the IVW MR estimate of each PCa single nucleotide polymorphism with cytokines versus 1/standard error (1/SEIV). (A–F) Adenosine deaminase, axin-1, CXCL6, Flt3L, IL-24, and IL-33. CXCL = C-X-C motif chemokine ligand, Flt3L = fms-related receptor tyrosine kinase 3 ligand, IL = interleukin, IVW = inverse variance weighted, MR = Mendelian randomization, PCa = prostate cancer.

The results of the primary MR study of 91 inflammatory cytokines were presented in Figure 4 and Table S4, Supplemental Digital Content, https://links.lww.com/MD/P839. The inference of the IVW method suggested that the progress of PCa was positive correlated with ascended abundance of adenosine deaminase (Beta: 1.7661, 95% CI: 0.2092–3.3229, *P* = .026), axin-1 (Beta: 1.9185, 95% CI: 0.1548–3.6822, *P* = .033), CXCL6 (Beta: 1.9681, 95% CI: 0.4207–3.5155, *P* = .013), Flt3L (Beta: 1.6589, 95% CI: 0.0733–3.2446, *P* = .040), IL-24 (Beta: 2.2091, 95% CI: 0.4682–3.9500, *P* = .013), IL-33 (Beta: 1.8438, 95% CI: 0.0815–3.6061, *P* = .040). Resemble estimation results were certificated by other analysis methods. Scatterplots and funnel plots of MR analyses of between PCa and adenosine deaminase, axin-1, CXCL6, IL-24, IL-33, and Flt3L are displayed in Figures 5 and 6.

Cochrane *Q* test assessment of IVW indicated that there is no conspicuous heterogeneity among the selected IVs and the MR-Egger analysis did not exhibit orient pleiotropy (Table S5, Supplemental Digital Content, https://links.lww.com/MD/P839). The results of the leave-one-out method identified the absence of significant outliers, indicating that no individual SNPs had an impact on the overall causal estimation (Figure S2, Supplemental Digital Content, https://links.lww.com/MD/P838). Particular SNPs information and statistical data and are organized in Table S6, Supplemental Digital Content, https://links.lww.com/MD/P839.

## 4. Discussion

This study employed a 2-sample MR analysis to investigate the potential effects of 91 inflammatory cytokines on PCa and conducted a reverse MR study to explore whether there is a reverse causal relationship. The findings suggested that Flt3L, MCP2, and MCP4 may be predisposing factors in the initiation of PCa, while adenosine deaminase, axin-1, CXCL6, IL-24, IL-33, and Flt3L in a downstream position in the pathogenesis of PCa. Elevation of Flt3L and MCP4 induced the development of PCa, while MCP2 might delay the progression of PCa. Notably, Flt3L was reflected in both the morbidity and downstream stages of PCa, indicating a bidirectional regulatory effect.

Intraprostatic inflammation is widely recognized as a risk factor for PCa development,^[26]^ contributing to prostate carcinogenesis through oxidative stress-induced reactive oxygen species generation, which promotes DNA mutagenesis.^[27]^ The abnormal proliferation of immune cells in the prostate tumor microenvironment will result in genetic mutation damage, which will promote tumor immune escape and induce tumorigenesis.^[28,29]^ Chronic inflammation may induce prostate adenocarcinoma to neuroendocrine PCa through epigenetic reprogramming. This is a rare and aggressive subtype, which is often accompanied by immunosuppressive infiltration of M2 macrophages and regulatory T-cells, and the significantly increased expression of programmed cell death ligand 1.^[30]^ Previous basic experimental research demonstrated that the elevated levels of cytokines such as IL-30, IL-6, TNF-α, and IL-17 could activate a range of phlogistic pathways, accelerating epigenetic variation and promoting PCa deterioration.^[31–33]^

PCa have many immune markers and been considered as one of the most suitable tumors for immunotherapy.^[34]^ In the past decade, immunotherapy has greatly changed the treatment pattern of malignant tumors, but immune checkpoint inhibitors are by no means a panacea. PCa also has the characteristics of hormone dependence, low tumor mutation load, and immunosuppressive microenvironment, which are different from other malignant tumors. Despite previous studies had performed relevance between inflammation and PCa, classical epidemiology and experimental research were susceptible to different imaginable confounders, which hinders the establishment of a direct causal nexus between inflammatory factors and PCa results. Genotypes and serum protein genomes were independent of confounding variables in the population, which could prevent the defect of conventional methods and improve the accuracy of analysis.^[24]^

It can be concluded from the forward MR analysis that Flt3L and MCP4 were the stimulus of PCa, while MCP2 acted as an inhibitor. MCP4 was a novel human β chemokine, which had a strong chemotactic effect on monocytes and eosinophils, inducing inflammation upon binding to CC chemokine receptor 3^[35,36]^ and exhibited low expression in PCa serum samples.^[37]^ The results of the present MR analysis concluded that MCP4 is a risk factor for PCa, and it has been proved that MCP4 has low expression in prostate patients. So further investigation should be made on the involvement of MCP4 in the pathogenesis of PCa based on the results of the present study and the previous studies, so as to formulate a targeted therapeutic regimen regarding MCP4 against PCa. In contrast, MCP2 exerted anti-tumor effects and immune monitoring activity by activating mast cells, eosinophils, basophils, monocytes, T-cells, and NK cells.^[38]^ The detection of these certain cytokines as latent interfering factors to PCa highlights their clinical value as drug targets.

In reverse MR analysis, the analysis results of adenosine deaminase, axin-1, CXCL6, Flt3L, IL-24, and IL-33 have statistical significance. The knockdown of adenosine deaminase was verified to suppress the proliferation and promoted apoptosis of PCa cells.^[39]^ In addition, IL-33 had significant impact on cancer immune surveillance in primary tumors and been proven to be related to recurrence of PCa,^[40]^ and the regulation of IL-33/ST2 signaling pathway might lead to invasion and metastasis of PCa.^[41]^ IL-24 is a member of the IL-10 gene family, performing multiple anti-tumor characters comprising specific involvement in regulating tumor microenvironment and modulation of immune responses.^[42–44]^ CXCL6 can potentially promote the proliferation of blood vessels in PCa and induce the transformation of PCa into a highly invasive, hormone independent state.^[45]^ By activating the axin-1/β-catenin signaling pathway, tryptophan could enhance tumor cell proliferation and multiple migration, leading to poor prognosis.^[46]^

Significantly, Flt3L played a dual regulatory role in the occurrence and development of PCa. As a notable immune regulatory protein in hematopoiesis, Flt3L could regulated the proliferation and differentiation of hematopoietic stem cells when interacting with the receptor FLT3,^[47]^ and might activate immune function by promoting dendritic cell maturation. Furthermore, Flt3L could restrain the proliferation in situ tumor and lymph node metastasis with the combination with other adjuvants. Therefore, we speculated that Flt3L, as an upstream cytokine for prostate carcinogenesis, is the basis for its ability to act as a cellular tumor adjuvant, while be regarded as a downstream cytokine for prostate carcinogenesis, Flt3L could inhibit local tumor infiltration and distant metastasis.^[48–50]^

These particular inflammatory cytokines have been identified as latent triggers of PCa, which emphasized their significance as viable immunotherapeutic targets. Promising research is being conducted on targets such as Flt3L and IL-24 in the treatment of PCa, but additional verification is necessitated to confirm their therapeutic efficacy.^[51,52]^ Consequently, exploring the interaction mechanisms between different inflammatory cytokines and their impact on triggering immune responses are crucial for researching the etiology and prognosis of PCa.

Despite MR analysis has advantages in avoiding reverse causal relationships, certain limitations are still worth considering. First, the GWAS summary data is mostly sourced from Europeans, which may hinder the extrapolation of research results and unable to eliminate potential bias in European population data. Second, different subtypes and clinical features of PCa may have inconsistent genetic variations, hence detailed sample stratification is needed to address the causal relationship between subgroups. Third, MR research has certain deficiency in identifying the complex mechanisms or biological pathways of PCa. Therefore, further experimental research and clinical trials in different races and districts are necessary to verify the associations discovered by MR research. Previous cohort studies have shown that tumor immune suppression is more pronounced in African populations with PCa and is associated with fatal PCa. This provides us with new possibilities and research directions for further research.^[53]^ In the next step, we propose conducting to include multi-ethnic GWAS data (such as UK Biobank Africa subgroup and East Asia biological bank) and use cross-ethnic meta-analysis or trans-ethnic MR methods to assess the robustness of our findings, which will help determine whether the observed causal effects are consistent across ethnic groups or are altered by the genetic structure of a particular population.

## 5. Conclusion

In conclusion, this study delved into the genetic relationship between inflammatory cytokines and PCa via two-sample MR analysis. The results implicated Flt3L, MCP2, and MCP4 as potential upstream contributors to PCa initiation, while Flt3L, adenosine deaminase, axin-1, CXCL6, IL-24, and IL-33 were positioned downstream in PCa pathogenesis. Specifically, Flt3L, emerged as a bidirectional regulator, affects both disease onset and later period. Collectively, these results advance understanding of inflammatory mediators in PCa etiology, highlighting novel biomarkers and therapeutic targets for drug development.

## Acknowledgments

The authors express their gratitude to the participants and investigators of the study. This study utilized publicly available genome-wide association study (GWAS) summary statistics from UK Biobank (https://www.ukbiobank.ac.uk). All datasets were fully anonymized, and no individual-level data were accessed. Ethical approval for the original GWAS studies was obtained by the respective data contributors and this secondary analysis of de-identified data does not require additional ethical review. The authors sincerely thank related investigators for sharing the GWAS summary statistics included in this study.

## Author contributions

**Conceptualization:** Yantong Wan.

**Data curation:** Yantong Wan.

**Funding acquisition:** Jieyan Wang, Hui Liang.

**Writing – original draft:** Jieyan Wang, Qi Cheng, Fangyu Luo, Yantong Wan, Hui Liang.

**Writing – review & editing:** Jieyan Wang, Qi Cheng, Fangyu Luo, Yantong Wan, Hui Liang.

## Supplementary Material


