# Supplementary material for: Investigating bidirectional causality between prostate cancer and inflammatory factors: A 2-sample Mendelian randomization analysis
Source: Medicine (Baltimore). 2025 Sep 5;104(36):e44180. doi: 10.1097/MD.0000000000044180 (PMC12419315; doi:10.1097/MD.0000000000044180)

Fig S1. Leave-one-out sensitivity analyses of Mendelian randomization analyses for Flt3L, MCP2 and MCP4 in PCa. (a -c)Flt3L, MCP2 and MCP4.

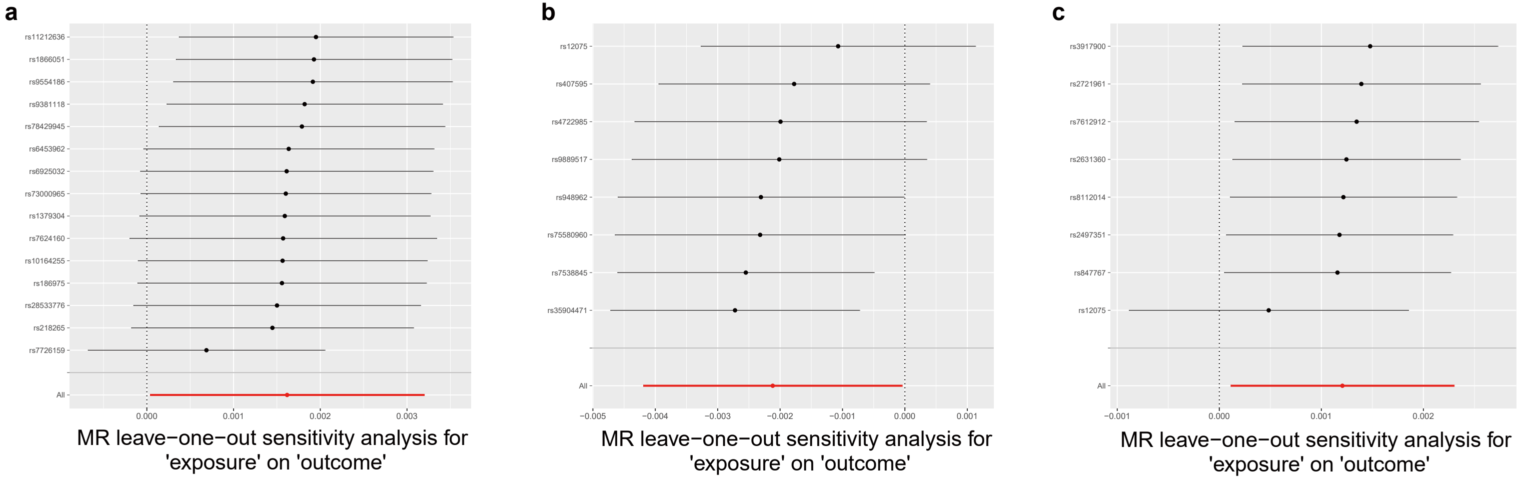

Fig S2. Leave-one-out sensitivity analyses of Mendelian randomization analyses between PCa and inflammatory cytokines. (a–f): Adenosine Deaminase, Axin-1, CXCL6, Flt3L, IL-24 and IL-33.

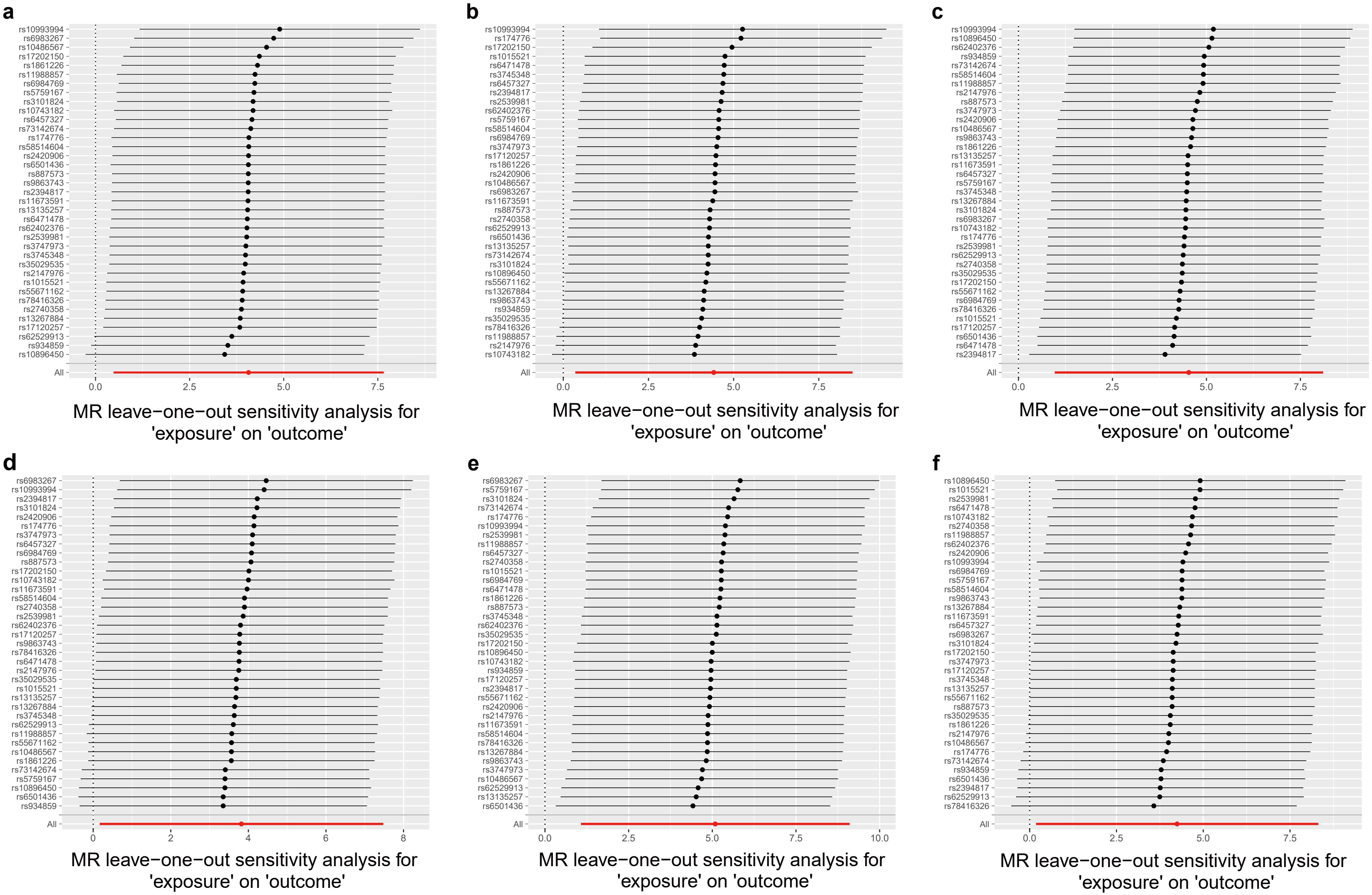

Supplement: Supplementary file 2 [file medi-104-e44180-s002.pdf]
